# Genome-Wide Identification of Homeodomain Leucine Zipper (HD-ZIP) Transcription Factor, Expression Analysis, and Protein Interaction of HD-ZIP IV in Oil Palm Somatic Embryogenesis

**DOI:** 10.3390/ijms24055000

**Published:** 2023-03-05

**Authors:** Kamolwan Khianchaikhan, Suvichark Aroonluk, Supachai Vuttipongchaikij, Chatchawan Jantasuriyarat

**Affiliations:** 1 Department of Genetics, Faculty of Science, Kasetsart University, Bangkok 10900, Thailand; 2 Program in Agriculture, Faculty of Agriculture and Life Sciences, Chandrakasem Rajabhat University, Bangkok 10900, Thailand; 3 Center for Advanced Studies in Tropical Natural Resources, National Research University-Kasetsart University (CASTNAR, NRU-KU), Bangkok 10900, Thailand; 4 Omics Center for Agriculture, Bioresources, Food and Health, Kasetsart University (OmiKU), Bangkok 10900, Thailand

**Keywords:** HD-ZIP, somatic embryogenesis, oil palm

## Abstract

Understanding the molecular mechanisms underlying somatic embryogenesis is essential for resolving the problems related to the long duration of the process and a low rate of somatic embryo induction in oil palm tissue culture. In this study, we conducted genome-wide identification of the oil palm homeodomain leucine zipper (EgHD-ZIP) family, which is one of the plant-specific transcription factors reported to be involved in embryogenesis. EgHD-ZIP proteins can be divided into four subfamilies, which have similarities in gene structure and protein-conserved motifs within a group. In silico expression analysis showed that the expression of *EgHD-ZIP* gene members in the *EgHD-ZIP I* and *II* families, as well as most members in the *EgHD-ZIP IV* family, were up-regulated during the zygotic and somatic embryo developmental stages. In contrast, the expression of *EgHD-ZIP* gene members in the *EgHD-ZIP III* family was down-regulated during zygotic embryo development. Moreover, the expression of *EgHD-ZIP IV* genes was validated in the oil palm callus and at the somatic embryo stages (globular, torpedo, and cotyledon). The results revealed that *EgHD-ZIP IV* genes were up-regulated at the late stages of somatic embryogenesis (torpedo and cotyledon). While BABY BOOM (BBM) gene was up-regulated at the early stage of somatic embryogenesis (globular). In addition, the Yeast-two hybrid assay revealed the direct binding between all members of the oil palm HD-ZIP IV subfamily (EgROC2, EgROC3, EgROC5, EgROC8, and EgBBM). Our findings suggested that the EgHD-ZIP IV subfamily and EgBBM work together to regulate somatic embryogenesis in oil palms. This process is important because it is widely used in plant biotechnology to produce large quantities of genetically identical plants, which can be used for oil palm tissue culture improvement.

## 1. Introduction

Somatic embryogenesis (SE) is an in vitro developmental process in which somatic cells can divide and differentiate into embryonic cells under certain conditions via the following stages: globular-shaped, heart-shaped, torpedo-shaped, and cotyledon stages, without gamete fusion. The main factors involving the induction of somatic embryogenesis depend on explant types, plant growth regulators, stress factors, and ectopic expression of identity genes [1,2]. Somatic embryogenesis is the most widely used approach for micropropagation in tissue culture, thereby serving as a key strategy for enhancing plant production.

The Homeodomain-leucine zipper protein (HD-ZIP) family is one of the plant-specific transcription factors reported to be involved in embryogenesis. HD-ZIPs contain a homeodomain (HD) and a leucine zipper motif (LZ). HD serves as a DNA binding domain. LZ, closely downstream of the HD, is involved in protein dimerization [3,4,5]. HD-ZIPs family is divided into the following four subfamilies: HD-ZIP I to HD-ZIP IV, based on the homology of HD-ZIP domain, additional conserved motif, gene structure, and function [5].

The HD-ZIP I subfamily contains a highly conserved HD and a less conserved LZ. At the C-terminus, it contains an additional AHA (aromatic and large hydrophobic residues in an acidic context) motif, which involves interacting with the basal transcription complex components [6,7]. ATHB5 is a member of HD-ZIP I, which expresses in the protoderm of an embryo and relates to auxin signaling during embryogenesis [8]. The HD-ZIP II subfamily contains highly conserved HD and LZ, and the following two additional domains: the CPSCE (Cys-Pro-Ser-Cys-Glu amino acids) and ZIBEL. The CPSCE domain plays a role as a redox sensor, and the ZIBEL domain is necessary for the interaction with BELL HD proteins [4,7,9]. Several HD-ZIP II proteins are reported to be involved in auxin transport during embryogenesis [10,11]. The HD-ZIP III and IV subfamilies contain an additional Steroidogenic acute regulatory protein-related lipid transfer (START) domain, followed by the Start-adjacent domain (SAD). The START domain is a lipid-binding domain and is necessary for nuclear transport [4,7,12]. Moreover, an additional MEKHLA (Met-Glu-Lys-His-Leu-Ala amino acids) domain is found in HD-ZIP III, which acts as a negative regulator by blocking its dimerization of the LZ domain. HD-ZIP III is post-transcriptionally regulated by miRNAs for embryogenesis [13]. HD-ZIP IV was reported to interact with BABY BOOM (BBM) to control cell proliferation during embryogenesis [14]. The *BBM* gene is one of the biomarkers for embryogenesis. Overexpression of *BBM* can induce somatic embryos in several plant species [15,16,17,18]. In contrast, co-overexpression of *BBM* and *HDG*, one member of HD-ZIP IV, shows a decrease in the percentages of somatic embryo induction, suggesting antagonistic functions between BBM and HDG [14].

Oil palm (*Elaeis guineensis*) is one of the oil crops mainly propagated by tissue culture. In general, oil palm tissue culture takes approximately one and a half years, from explants to plantlets. The callus formation takes 2–4 months, and the somatic embryogenesis takes another 7–8 months [19]. In oil palm tissue culture, however, there is a long time requirement for somatic embryogenesis, and a low rate of somatic embryo induction [20,21]. A previous study shows that *EgBBM* has high expression during oil palm somatic embryogenesis [22]. Additionally, *Arabidopsis* HD-ZIP IV interacts with the embryogenic marker, BBM, as demonstrated by yeast two-hybrid assay. The results indicate that BBM and HD-ZIP IV together control somatic embryogenesis [14]. However, the HD-ZIP protein family in oil palm has not yet been identified and characterized. In this study, our focus was on the genome-wide identification of the oil palm HD-ZIP family, a comprehensive study of HD-ZIP IV expression during oil palm somatic embryogenesis, and the interaction between HD-ZIP IV and BBM.

## 2. Results

### 2.1. Phylogenetic Analysis and Chromosome Location of Oil Palm HD-ZIPs Family

**Figure 1:**
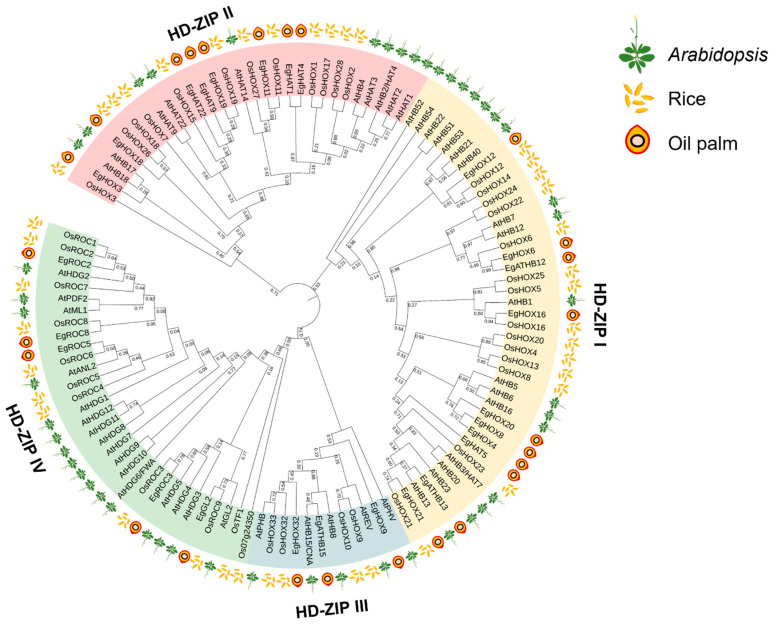
Phylogenetic analysis of HD-ZIP proteins in oil palm, *Arabidopsis*, and rice. The phylogenetic tree was constructed based on HD-ZIP protein sequences, which compose of 26 oil palm HD-ZIP proteins, 48 *Arabidopsis* HD-ZIP proteins, and 41 rice HD-ZIP proteins. Four HD-ZIP subfamilies are represented by different colors, including HD-ZIP I (yellow), HD-ZIP II (pink), HD-ZIP III (blue), and HD-ZIP IV (green).

Forty-eight HD-ZIP protein sequences of *Arabidopsis*, forty-one sequences of rice, and twenty-six sequences of oil palm were identified and downloaded. A phylogenetic tree was constructed to study the relationship between HD-ZIP proteins of *Arabidopsis*, rice, and oil palm. The HD-ZIP protein family could be divided into four subfamilies (HD-ZIP I–IV). The HD-ZIP I subfamily is composed of 17 *Arabidopsis* proteins, 14 rice proteins, and 10 oil palm proteins. The HD-ZIP II subfamily is composed of 10 *Arabidopsis* proteins, 12 rice proteins, and 8 oil palm proteins. The HD-ZIP III subfamily is composed of 5 *Arabidopsis* proteins, 4 rice proteins, and 3 oil palm proteins. The HD-ZIP IV subfamily is composed of 16 *Arabidopsis* proteins, 11 rice proteins, and 5 oil palm proteins. The number of HD-ZIPs in oil palm was less than in *Arabidopsis* and rice. The HD-ZIP I and HD-ZIP III subfamilies consisted of the largest and smallest members, respectively. The oil palm HD-ZIP I and II subfamilies were more closely related to rice than *Arabidopsis*, whereas the oil palm HD-ZIP III and IV subfamilies were closely related to both *Arabidopsis* and rice (Figure 1).

**Figure 2:**
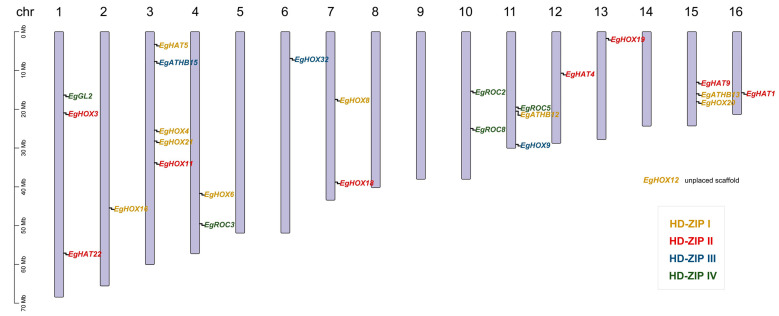
Chromosome location of oil palm *EgHD-ZIP* genes. *EgHD-ZIP* genes were mapped to twelve oil palm chromosomes. HD-ZIP subfamilies are represented by different colors. Chromosome numbers were displayed at the top of each chromosome.

An examination of the oil palm HD-ZIP (*EgHD-ZIP*) gene distribution on oil palm chromosomes found that they were not evenly distributed across all sixteen oil palm chromosomes (Figure 2). Twenty-five *EgHD-ZIP* genes were mapped to twelve chromosomes, including chromosomes 1, 2, 3, 4, 6, 7, 10, 11, 12, 13, 15, and 16, while the remaining chromosomes had no *EgHD-ZIP* gene. Chromosome 3 had the largest number, with five *EgHD-ZIP* genes. *EgGL2*, *EgHOX3*, and *EgHAT22* were located on chromosome 1. *EgHOX16* was located on chromosome 2. *EgHAT5*, *EgATHB15*, *EgHOX4*, *EgHOX21*, and *EgHOX11* were located on chromosome 3. *EgHOX6* and *EgROC3* were located on chromosome 4. *EgHOX32* was located on chromosome 6. *EgHOX8* and *EgHOX18* were located on chromosome 7. *EgROC2* and *EgROC8* were located on chromosome 10. *EgROC5*, *EgATHB12*, and *EgHOX9* were located on chromosome 11. *EgHAT4* was located on chromosome 12. *EgHOX19* was located on chromosome 13. *EgHAT9*, *EgATHB13*, and *EgHOX20* were located on chromosome 15. *EgHAT1* was located on chromosome 16. However, *EgHOX12* was unplaced on the chromosome scaffold (Figure 2).

### 2.2. Gene Structure and Conserved Motif of Oil Palm HD-ZIPs Family

**Figure 3:**
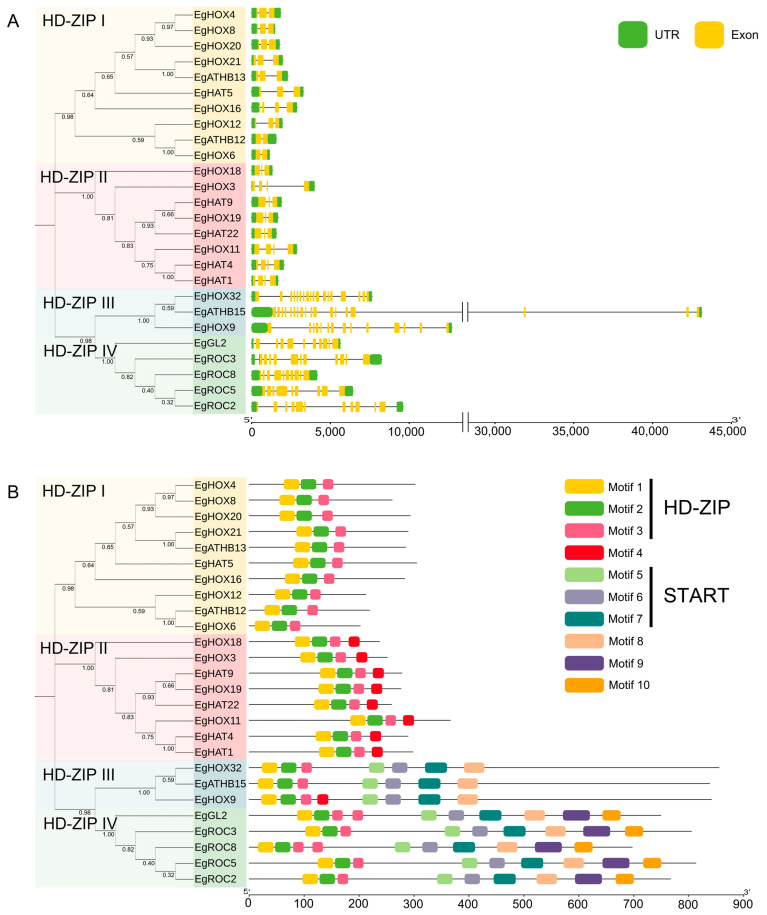
Gene structures and conserved motifs of oil palm *EgHD-ZIP* genes. (**A**) Gene structure of *EgHD-ZIP* genes. Green and yellow boxes indicate UTRs and CDSs, respectively. Black lines indicate introns. (**B**) Conserved motifs of EgHD-ZIPs. Ten conserved motifs are represented in different color boxes. Motifs 1–3 represent the homeodomain leucine zipper (HD-ZIP) and motifs 5–7 represent the START domain.

The structure of *EgHD-ZIP* genes was determined according to the oil palm genome annotation. The results revealed a similar pattern in exon–intron profiles, which were closely related within their group (Figure 3A). *EgHD-ZIP* genes in HD-ZIP I subfamily contain 2 to 4 exons. *EgHD-ZIP* genes in HD-ZIP II subfamily contain 3 to 4 exons. *EgHD-ZIP* genes in HD-ZIP III subfamily contain the highest number of exons, with 14 (*EgHOX9*) and 18 (*EgHOX32* and *EgATHB15*) exons. *EgHD-ZIP* genes in the IV subfamily contain 9 to 11 exons. Gene lengths in subfamily III were notably longer than those in subfamilies I, II, and IV.

Furthermore, the amino acid sequences of 26 oil palm HD-ZIP proteins were analyzed. Ten conserved motifs were identified. Motifs 1–3 corresponded to the HD-ZIP, which were found and highly conserved in all four subfamilies. Motif 4 was found only in the HD-ZIP II subfamily, along with HOX9 from the HD-ZIP III subfamily. Motifs 5–7 corresponded to the START domain, which was found in subfamilies III and IV. HD-ZIP IV was the only subfamily that contained motif 9–10 (Figure 3B). The exon–intron structures and conserved-motif characteristics of oil palm HD-ZIPs were common among members of the same subfamily.

**Figure 4:**
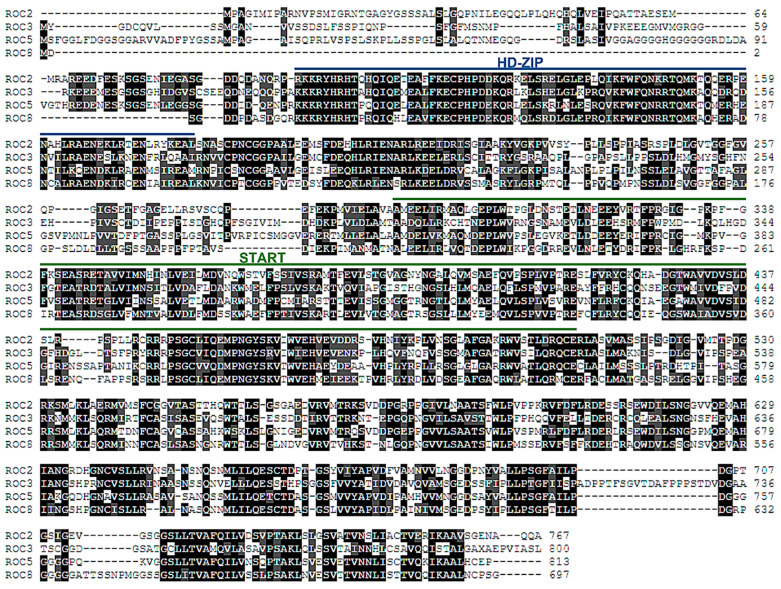
Protein sequence analysis of the oil palm EgHD-ZIP IV family. Multiple protein sequence alignment was performed using the Clustal Omega, and colored alignment with 70% identity was performed using Colour Align Conservation. Identical amino acids were colored in black, and similar amino acids were colored in gray. Blue and green bars indicated HD-ZIP and START domains, respectively.

The *EgROC2*, *EgROC3*, *EgROC5*, and *EgROC8* genes of the HD-ZIP IV subfamily were cloned and sequenced from oil palm materials in this study. The *EgROC2*, *EgROC3*, *EgROC5*, and *EgROC8* genes encoded 767, 800, 813, and 697 amino acids, respectively. The HD-ZIP domain resided at the amino acid positions 96–181, 93–178, 124–209, and 15–100 of EgROC2, EgROC3, EgROC5, and EgROC8, respectively. The START domain resided at the amino acid positions 293–506, 297–517, 338–557, and 215–434 of EgROC2, EgROC3, EgROC5, and EgROC8, respectively (Figure 4). This result indicated that EgROCs, members of the EgHD-ZIP IV family, shared a conserved gene structure and protein motif.

### 2.3. In Silico Expression Analysis of EgHD-ZIP Genes during Oil Palm Zygotic and Somatic Embryogenesis

**Figure 5:**
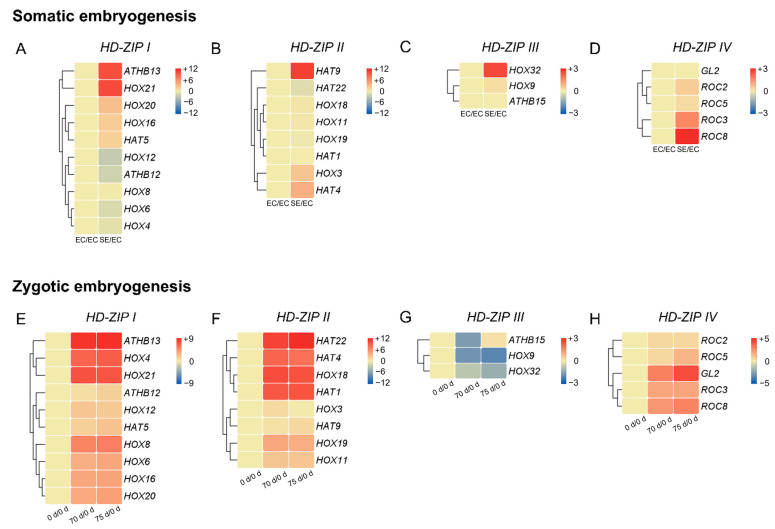
In silico expression analysis of *EgHD-ZIP* genes during oil palm zygotic and somatic embryogenesis. Heat map represents Log_2_FC of FPKM somatic embryo (SE)/embryogenic callus (EC) for somatic embryogenesis and Log_2_FC of FPKM (70 d/0 d and 75 d/0 d) for zygotic embryogenesis of *EgHD-ZIP I* (**A**,**E**), *II* (**B**,**F**), *III* (**C**,**G**), and *IV* (**D**,**H**) genes. The 0 d, 70 d, and 75 d indicate the day of zygotic embryogenesis during seed germination. Red color indicates up-regulation and blue color indicates down-regulation.

The in silico expression of *EgHD-ZIP* genes was analyzed, and the results were represented in a heatmap with the Log2FC of FPKM between control and embryo stages (Figure 5). The *EgHD-ZIP* subfamily I members *EgATHB13*, *EgHOX21*, *EgHOX20*, *EgHOX16*, and *EgHAT5* were up-regulated, but *EgHOX12*, *EgATHB12*, *EgHOX6*, and *EgHOX4* were down-regulated in the somatic embryo stage, and *EgHOX8* was not changed during the embryogenic callus stage (Figure 5A). The *EgHD-ZIP* subfamily II members *EgHAT9*, *EgHOX3*, and *EgHAT4* were up-regulated, but *EgHAT22* was slightly down-regulated in the somatic embryo stage, and *EgHOX18*, *EgHOX11*, *EgHOX19*, and *EgHAT1* were not changed (Figure 5B). The *EgHD-ZIP* subfamily III members *EgHOX32* and *EgHOX9* were up-regulated, but *EgATHB15* was not changed during the embryogenic callus stage (Figure 5C). All the *EgHD-ZIP* subfamily IV members exhibited a high expression level in the somatic embryo stage, except *EgGL2,* which was not expressed (Figure 5D). Interestingly, the *EgHD-ZIP I*, *II*, and *IV* members shared similar expression patterns in the somatic and zygotic embryogenesis, whereas the *EgHD-ZIP III* members showed the opposite pattern. *EgHox32* was up-regulated in the somatic embryo, but was down-regulated in the zygotic embryo (Figure 5E–H).

### 2.4. Validation of EgHD-ZIP IV Subfamily Gene Expression during Somatic Embryogenesis

**Figure 6:**
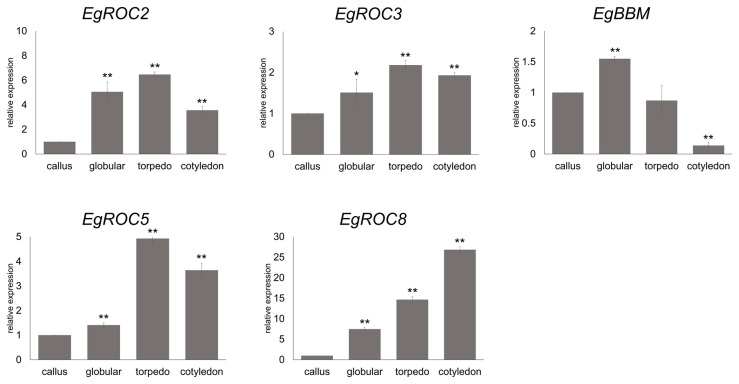
Validation of *EgHD-ZIP IV* and *EgBBM* gene expression during oil palm somatic embryogenesis. The relative expression level of *EgROC2*, *EgROC3*, *EgROC5*, *EgROC8*, and EgBBM genes in callus, globular, torpedo, cotyledon, and plantlet stages were calculated using the 2^−ΔΔCt^ method and presented as mean ± SD of three biological replicates. Asterisks indicate a significant difference from the callus stage (Student’s *t*-test: *—*p* < 0.05; **—*p* < 0.01).

A previous report reveals that HD-ZIP IV coordinates with BBM to control somatic embryogenesis in *Arabidopsis*. The expression of these genes was validated in the embryogenic callus and in three stages of the somatic embryo, using quantitative real-time PCR. The result showed that *EgROC2*, *EgROC3*, *EgROC5*, and *EgROC8* were expressed in the somatic embryo. All four genes were notably up-regulated at the late stages of somatic embryogenesis (torpedo and cotyledon) (Figure 6). Additionally, *EgBBM* was also found to be up-regulated during somatic embryogenesis, especially in the early stage of somatic embryogenesis (globular) (Figure 6).

### 2.5. Oil Palm HD-ZIP IV Proteins and BBM Interaction in Yeast-Two Hybrid Assay

**Figure 7:**
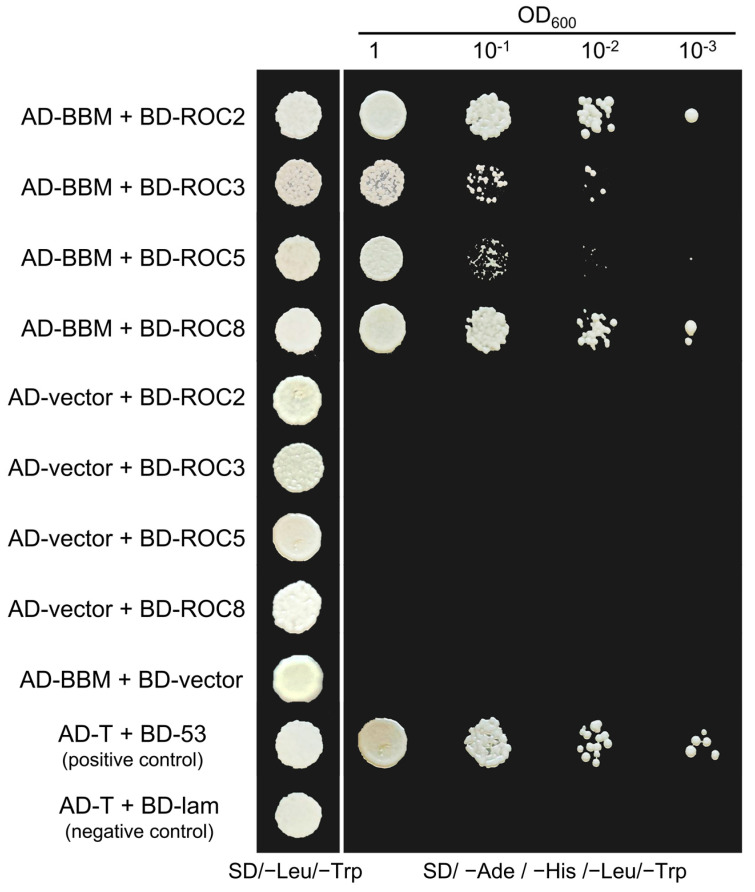
Yeast-two hybrid assay for EgHD-ZIP IV proteins and EgBBM interactions. EgROC2, EgROC3, EgROC5, and EgROC8 interacted with EgBBM. Transformed yeast cells were grown on SD/−Trp/−Leu and SD/−Trp/−Leu/−His/−Ade media. AD and BD indicated activation domain and binding domain fused with proteins, respectively.

Yeast two-hybrid assay was performed to investigate the interaction between oil palm BBM and members of the oil palm HD-ZIP IV subfamily. The result revealed that BBM interacted with the following members of the oil palm HD-ZIP IV subfamily: ROC2, ROC3, ROC5, and ROC8 (Figure 7).

## 3. Discussion

The HD-ZIP protein family is one of the plant-specific transcription factors reported to be involved in embryogenesis in several plant species [10,11,12,13,14]. However, the oil palm HD-ZIP protein family was rarely investigated. Therefore, a genome-wide identification and characterization of 26 oil palm *HD-ZIP* genes was performed in this study. All *EgHD-ZIP* genes encoded proteins contained conserved HD and LZ domains that acted as transcriptional regulators. In order to study the relationship between oil palm, rice, and *Arabidopsis*, the phylogenetic tree was constructed using EgHD-ZIPs, along with OsHD-ZIPs and AtHD-ZIPs. The phylogenetic relationship indicated the conservation of members in this protein family and revealed the close relationship between monocot and dicot species. Oil palm and rice HD-ZIP proteins, as a monocot group, shared higher similarities than those between oil palm and *Arabidopsis*. Moreover, the gene structure and conserved motif of HD-ZIPs family in soybean [23], grape [24], potato [25], pepper [26], and pineapple [27] were also conserved and closely related in a subfamily, such as EgHD-ZIPs [28,29].

Based on the transcriptomic data, nineteen and twenty-three *EgHD-ZIP* genes were differentially expressed during somatic and zygotic embryogenesis, respectively. The *EgHOX20* gene, a member of the HD-ZIP I family, is closely related to ATHB5, and a previous report revealed that auxin signaling is indirectly influenced by ATHB5 during embryonic development [8]. Additionally, the *EgHAT4* gene, a member in HD-ZIP II family, was an ortholog of ATHB2, which is an early auxin-inducible gene expressed in *Arabidopsis* embryos [10,30]. ATHB2 is controlled by the Phytochrome interacting factors 4 (PIF4) transcription factor, which promotes plant growth and development by activating the auxin-responsive gene [10,31,32]. *EgPIF4* was highly expressed during the entire development of somatic embryos (globular, torpedo, and cotyledon stages), and was previously reported to directly regulate *ATHB2* gene expression during embryo development through the auxin signaling pathway [33]. Other *HD-ZIP II* genes (*ATHB4* and *HAT3*) were expressed during embryogenesis and are required for correctly transporting auxin for embryo development in *Arabidopsis* [10]. *EgHAT4*, closely related to ATHB2, ATHB4, and HAT3, may play an auxin-mediated signaling role in embryogenesis, since it is up-regulated in the oil palm somatic and zygotic embryo stages. Moreover, *EgHOX18*/*EgHOX1* in the HD-ZIP II subfamily was also expressed at the early stages of somatic embryogenesis [34]. Additionally, *EgHOX32*, a member of the HD-ZIP III family, was expressed during oil palm somatic embryogenesis. *EgHOX32* is closely related to AtPHB, which expresses at the embryo stages in *Arabidopsis*. Several studies have demonstrated that *AtPHB* is recognized and regulated by miRNA165/166, which is needed for normal embryo development. Mutation of the PHB gene prevents recognition and regulation by miRNA165/166, leading to embryo defect [13,35,36]. Through the auxin signaling pathway, PHB also directly regulates the *LEC2* gene, which controls embryogenesis [36,37,38]. Nevertheless, somatic and zygotic embryos showed distinct patterns of *EgHD-ZIP III* gene expression. Consequently, auxin is described to be involved in embryogenesis through a complex regulatory network of auxin-responsive genes, including HD-ZIPs, in several plant species [39,40,41,42].

Several *HD-ZIP IV* genes are expressed during embryo development, and are associated with meristematic-related gene depletion in *Arabidopsis* [14]. Transcriptomic data showed that *EgHD-ZIP IV* genes exhibited transcript levels at various stages of oil palm zygotic and somatic embryo stages. *EgHD-ZIP IV* gene expression levels were validated in callus, globular, torpedo, and cotyledon, indicating that these genes were expressed during somatic embryogenesis, especially in the late stage of somatic embryogenesis. HD-ZIP IV proteins are reported to be presented in the outermost layer (L1) of an embryo and play an important role in stimulating cell differentiation [14,43,44]. Therefore, *EgHD-ZIP IV* genes might be related to cell differentiation during somatic embryogenesis. HD-ZIP IV proteins are known to interact with BBM, a biomarker for somatic embryogenesis, to regulate cell proliferation during embryo development in *Arabidopsis*. *EgBBM* was highly expressed in early somatic embryo stages, whereas *EgHD-ZIP IV* genes were highly expressed in late somatic embryo stage. *EgBBM* and *EgHD-ZIP IV* genes revealed an antagonistic expression, which may work together to regulate somatic embryogenesis.

Consistent with the previous report in *Arabidopsis*, the EgHD-ZIP IV members EgROC2, ROC3, EgROC5, and EgROC8 interacted with EgBBM. Overexpression of BBM can induce somatic embryos. In contrast, the down-regulation of multiple HDG genes can promote somatic embryos in *Arabidopsis*. Moreover, co-overexpression of AtBBM and AtHDG demonstrates an antagonistic function that decreases somatic embryo induction [14]. EgBBM and the EgHD-ZIP IV members may therefore work together to control cell proliferation and cell differentiation during oil palm somatic embryogenesis.

## 4. Materials and Methods

### 4.1. Plant Materials

Zygotic embryos of Tenera oil palm were used as explants to induce somatic embryos via an intermediate callus formation, based on Thuzar et al. (2011) [19]. The samples were collected at the following developmental stages: callus, and the somatic embryo at globular, torpedo, and cotyledon stages for the experiments.

### 4.2. Identification, Chromosome Location, and Gene Structure of Oil Palm HD-ZIP Gene Family

HD-ZIP protein sequences of *Arabidopsis* and rice [45] were downloaded and used to identify *EgHD-ZIP* genes by BLASTP search program against the oil palm genome (http://ncbi.nlm.nih.gov/genome/2669, accessed on 22 August 2021). *EgHD-ZIP* genes were mapped on chromosomes according to the oil palm genome annotation. The chromosome location of *EgHD-ZIP* genes was visualized by TBtools software (https://github.com/CJ-Chen/TBtools, accessed on 2 September 2022) [46]. Gene structure of *EgHD-ZIP* genes was determined according to the oil palm genome annotation. Protein conserved motifs were identified using MEME (http://meme-suite.org/tools/meme, accessed on 2 September 2022) tools. Gene structures and protein conserved motifs were visualized by Tbtools software.

### 4.3. Phylogenetic Relationship

HD-ZIP protein sequences of *Arabidopsis*, rice, and oil palm were aligned using ClustalW. A phylogenetic tree was constructed to study the relationship between HD-ZIP proteins of *Arabidopsis*, rice, and oil palm using the neighbor-joining (NJ) method with 1000 bootstrap values. HD (PF00046) and LZ (PF02183) domains were confirmed in EgHD-ZIP protein sequences using Pfam tools (pfam.xfam.org, accessed on 2 September 2022).

### 4.4. In Silico Expression Analysis of EgHD-ZIP Genes during Oil Palm Somatic Embryogenesis

RNA-seq data of oil palm zygotic embryogenesis (0 d—the zygotic embryo of non-germinated seeds; 70 d and 75 d—the zygotic embryo of germinated seeds as the early and late cotyledon stages, respectively) and somatic embryogenesis (EC—embryogenic callus; SE—somatic embryo) from oil palm tissue culture were downloaded from the Sequence Read Archive (SRA) database of NCBI of bioprojects PRJNA553301 and PRJNA699335, respectively [47]. The paired-end reads from each sample were aligned to the oil palm EG5.1 genome as the oil palm reference genome using TopHat version 2.1.1. The aligned reads were assembled, and the fragments per kilobase of transcript per million fragments mapped (FPKM) were calculated as expression levels using Cufflinks. Heatmap analysis was performed and visualized using Tbtools software, with Log_2_FC of FPKM (70 d/0 d and 75 d/0 d) for zygotic embryogenesis and Log_2_FC of FPKM (SE/EC) for somatic embryogenesis.

### 4.5. Quantitative Gene Expression Analysis of EgHD-ZIP IV Genes and EgBBM during Oil Palm Somatic Embryogenesis

Total RNA was extracted from oil palm embryogenic culture at different developmental stages, including embryogenic callus, and at the stages of the somatic embryo (globular, torpedo, cotyledon, and plantlet stage) using Spin Plant RNA (STRATEC Molecular, Berlin, Germany). RNA quantity and quality were assessed using Nanodrop (Thermo Scientific, Waltham, MA, USA) and agarose gel electrophoresis. Of total RNA, 1 µg was converted to cDNA using the Verso cDNA kit (Thermo Scientific, Waltham, MA, USA).

The cDNA samples were diluted and 50 ng of cDNA was used as a template for real-time qPCR with *EgHD-ZIP IV* and *EgBBM*-specific primers (Table S1, Supplementary Materials). Real-time qPCR was performed by the KAPA SYBR^®^ FAST qPCR Master Mix (2X) (Kapa Biosystems; Wilmington, MA, USA). The PCR reaction of *EgHD-ZIP IV* was performed as follows: 45 cycles at 95 °C for 15 s, 58 °C for 15 s, and 70 °C for 1 min. The PCR reaction of *EgBBM* was performed as follows: 45 cycles at 95 °C for 15 s, 60 °C for 15 s, and 70 °C for 1 min. *EgHD-ZIP IV* and *EgBBM* gene expression were normalized with the oil palm elongation factor gene (*EgEf1-α*) (NCBI accession number XM_019850296) (Table S1). The experiment was performed with three biological and three technical replicates. The 2^−ΔΔCt^ method was used to calculate the relative expression level of *EgHD-ZIP IV* and *EgBBM* genes. The Student’s t-test and one-way ANOVA were used to analyze significance, followed by Tukey’s test.

### 4.6. Cloning of EgHD-ZIP IV Genes and Sequence Analysis

The cDNA from the plantlet stage was used as a template to amplify coding sequences of *EgROC2*, *EgROC3*, *EgROC5*, and *EgROC8* genes with specific primers (Table S1). The PCR reaction of these genes was performed as follows: 35 cycles at 98 °C for 10 s, then at 60 °C (*EgROC2*), 62 °C (*EgROC3*), 59 °C (*EgROC5*), and 60 °C (*EgROC8*) for 45 s, and then at 72 °C for 1 min, followed by a final extension at 72 °C for 5 min. Coding sequences of *EgROCs* genes were cloned into the pGEM^®^-T Easy vector (Promega; Fitchburg, WI, USA). The positive clones were obtained for DNA sequencing (Barcode-tagged sequencing service, U2Bio; Bangkok, Thailand). A translational prediction of the coding sequences of *EgROCs* genes was performed using EMBOSS Transeq (http://ebi.ac.uk/Tools/st/emboss_transeq/, accessed on 2 September 2022). Multiple alignments of EgROCs protein sequences were conducted using Clustal Omega (https://www.ebi.ac.uk/Tools/msa/clustalo/, accessed on 2 September 2022), and the colored alignment with 70% identity agreement was conducted using Colour Align Conservation (https://www.bioinformatics.org/sms2/color_align_cons.html, accessed on 2 September 2022).

### 4.7. Binding between EgHD-ZIP IV and EgBBM

Yeast two-hybrid assay was performed with the Yeastmaker Yeast Transformation System2 (Clontech; Mountain View, CA, USA). Coding sequences of *EgHD-ZIP IV* genes were cloned into the pGBKT7 vector and transformed to Y2Hgold yeast stain. This transformed yeast strain was selected using a SD/−Trp medium. The coding sequence of *EgBBM* gene was cloned into pGADT7 vector and transformed into the Y187 yeast strain. This transformed yeast strain was selected using a SD/−Leu medium. Then, the colonies from both transformed yeast strains were used to co-culture for yeast mating and selected using a SD/−Leu/−Trp medium. Next, a SD/−Leu/−Trp/−His/−Ade medium was used to investigate protein interaction. The pGBKT7-53 and pGADT7-T vectors were used as a positive control, while the pGBKT7-Lam and pGADT7-T vectors were used as a negative control.

## 5. Conclusions

*HD-ZIP* genes have an important role in embryo development. In this study, the HD-ZIP family was identified across the entire genome of oil palm. A total of 26 identified *EgHD-ZIP* genes were divided into four subfamilies based on their similarities in gene structures and conserved protein motifs. Their expression profiles are displayed during somatic and zygotic embryogenesis processes. Furthermore, *EgHD-ZIP IV* genes might contribute to oil palm somatic embryogenesis, as their expression patterns are the opposite of *EgBBM*, an embryogenesis-marker gene. Moreover, all members of EgHD-ZIP IV interacted with EgBBM, suggesting that they work together to regulate oil palm somatic embryogenesis. These findings provided fundamental information on the molecular mechanism of *EgHD-ZIP* genes involved in oil palm somatic embryogenesis. Manipulation of EgHD-ZIPs and BBM expression during oil palm tissue culture will facilitate and accelerate somatic embryogenesis during oil palm tissue culture.

## Data Availability

No new data were created or analyzed in this study. Data sharing is not applicable to this article.

## Supplementary Materials

The following supporting information can be downloaded at: https://www.mdpi.com/article/10.3390/ijms24055000/s1.
